# Standard of care for COVID-19 in randomized clinical trials registered in trial registries and published in preprint servers and scholarly journals: a cross-sectional study

**DOI:** 10.1186/s12874-022-01646-1

**Published:** 2022-06-17

**Authors:** Mahir Fidahic, Danijela Nujic, Marta Civljak, Renata Runjic, Filipa Markotic, Marin Vidak, Livia Puljak

**Affiliations:** 1 Faculty of Medicine, University of Tuzla, Tuzla, Bosnia and Herzegovina; 2 Department of Public Health, Faculty of Medicine, Osijek, Croatia; 3 Center for Evidence-Based Medicine and Health Care, Catholic University of Croatia, Ilica 242, 10000 Zagreb, Croatia; 4 University of Split School of Medicine, Split, Croatia; 5 Medical School, University of Mostar, Mostar, Bosnia and Herzegovina

**Keywords:** SARS-CoV-2, COVID-19, Coronavirus, Original research, Clinical trial, Standard of care

## Abstract

**Background:**

The concept of standard of care (SoC) treatment is commonly utilized in clinical trials. However, in a setting of an emergent disease, such as COVID-19, where there is no established effective treatment, it is unclear what the investigators considered as the SoC in early clinical trials. The aim of this study was to analyze and classify SoC reported in randomized controlled trial (RCT) registrations and RCTs published in scholarly journals and on preprint servers about treatment interventions for COVID-19.

**Methods:**

We conducted a cross-sectional study. We included RCTs registered in a trial registry, and/or published in a scholarly journal, and/or published on preprint servers medRxiv and bioRxiv (any phase; any recruitment status; any language) that aim to compare treatment interventions related to COVID-19 and SoC, available from January 1, 2020, to October 8, 2020. Studies using „standard“ treatment were eligible for inclusion if they reported they used standard, usual, conventional, or routine treatment. When we found such multiple reports of an RCT, we treated those multiple sources as one unit of analysis.

**Results:**

Among 737 unique trials included in the analysis, 152 (21%) reported that SoC was proposed by the institutional or national authority. There were 129 (18%) trials that reported component(s) of SoC; the remaining trials simply reported that they used SoC, with no further detail. Among those 129 trials, the number of components of SoC ranged from 1 to 10. The most commonly used groups of interventions in the SoC were antiparasitics (62% of the trials), antivirals (57%), antibiotics (31%), oxygen (17%), antithrombotics/anticoagulants (14%), vitamins (13%), immunomodulatory agents (13%), corticosteroids (12%), analgesics/antipyretics (12%). Various combinations of those interventions were used in the SoC, with up to 7 different types of interventions combined. Posology, timing, and method of administration were frequently not reported for SoC components.

**Conclusion:**

Most RCTs (82%) about treatment for COVID-19 that were registered or published in the first 9 months of the pandemic did not describe the “standard of care” they used. Many of those interventions have, by now, been shown as ineffective or even detrimental.

**Supplementary Information:**

The online version contains supplementary material available at 10.1186/s12874-022-01646-1.

## Background

Soon after the outbreak of COVID-19, thousands of publications ensued and thousands of clinical trials about COVID-19 were registered [1]. In our earlier study, we noticed that many registered clinical trials for COVID-19 mentioned standard of care (SoC) as a comparator for an intervention tested for COVID-19, but without an explanation/description of what will be the SoC [1].

The concept of SoC (also called standard care; standard treatment; usual care, basic care, conventional treatment) is well known and utilized in clinical trials. It includes providing the highest attainable, the best currently available, proven treatment, established effective treatment [2]. In a randomized controlled trial (RCT), a new therapeutic intervention can be compared to the SoC. Mostly, the SoC should be evidence-based with confirmed specific criteria based on knowledge, indications, outcomes, and requirements [3].

However, in a setting of an emergent disease, such as COVID-19, where there is no established effective treatment, it is unclear what the investigators considered as the SoC.

The aim of the study was to analyze and classify SoC mentioned in RCTs registered in trial registries and RCTs published in scholarly journals and on preprint servers about treatment interventions for COVID-19.

## Methods

### Study design

This was a cross-sectional study.

### Protocol and registration

We defined a protocol for this review prospectively, and we published it on Open Science Framework (https://osf.io/he9c8/) after the final draft acceptance by all co-authors and before the start of any work.

### Eligibility criteria

We included RCTs registered in a trial registry, and/or published in a scholarly journal and/or published on preprint servers medRxiv and bioRxiv that aim to compare treatment interventions related to COVID-19 and SoC, available from January 1, 2020, to October 8, 2020. Any type of treatment intervention was eligible. Trials in any clinical development phase were eligible. Trial registrations in any recruitment status were eligible, i.e. regardless of whether they were labeled as not recruiting, recruiting, completed. We did not use any language restrictions, i.e. we aimed to include trial registrations, articles and preprints published in English or other languages. Studies using SoC were eligible for inclusion if they reported they used standard, usual, conventional, or routine treatment.

We excluded all types of non-randomized studies, including quasi-randomized trials. We excluded studies marked as canceled (withdrawn).

When we had found RCT protocols published in preprint servers or scholarly journals, we tried to identify whether these RCT protocols were also registered in included clinical trial registers and whether they had been published in our included completed RCTs. We used clinical trial registration identifiers to recognize multiple reports of a single RCT. When we found such multiple reports of an RCT, we treated those multiple sources as one unit of analysis. For trials with multiple sources of information (one or more registrations, preprint, full-text), we included information from the most informative source, which was defined as the one with the most elaborate description of SoC. We excluded overlapping trials between analyzed information sources. Detailed methodology for analysis of overlap is shown in Supplementary file 1 (https://osf.io/he9c8/).

### Information sources

To retrieve registered clinical trials, we downloaded all records of COVID-19 trials from the WHO International Clinical Trials Registry Platform (ICTRP) [4]. To retrieve RCTs published in scholarly journals, we searched Cochrane COVID-19 Study Register freely available online with filters „Journal article“ and „Randomised“. Cochrane COVID-19 Study Register is a freely-available, continually updated, annotated reference collection of studies on COVID-19. Data sources for the Register include weekly searches of PubMed and Embase, among multiple information sources searched.

For RCTs published on preprint servers medRxiv and bioRxiv, we searched the COVID-19 Portfolio tool by the National Institutes of Health (https://icite.od.nih.gov/covid19/search/).

### Screening of studies

The eligibility of each potentially relevant registered or published trial was screened by two review authors independently for eligibility (by three pairs of authors: MF and DN, MC and RR, FM and MV). Disagreements were resolved via discussion or consultation with the third author (LP).

### Data charting process

One author extracted data (MF, MC, FM participated in this step), and another author verified all extractions (DN, RR, MV participated in this step). For trials registered in trial registries, we extracted the following data: registration number, trial registry name, date when the registration was first posted, type of participants (inclusion criteria; whether patients were hospitalized or outpatients), intervention, if the authors mention they used standard of care/standard of therapy (yes/no), description of components of SoC (extracted verbatim), description of dose in the SoC, description of a regimen of SoC, sponsor name, sponsor country, recruitment status (recruiting, not yet recruiting, withdrawn, published, etc.), number of participants planned, the country where the study will be conducted (using the whole count method was used, with each country counted once, regardless of the number of sites from an individual country).

For published RCT, we extracted the following data: journal name, 2019 Journal Impact Factor (JIF), type of participants (inclusion criteria, whether patients were hospitalized or outpatients), intervention, outcomes, if the authors mention they used SoC (yes/no), description of components of SoC (extracted verbatim), description of dose in the SoC, description of a regimen of SoC, sponsor name, sponsor country, number of participants.

### Synthesis of results

We analyzed data using descriptive statistics, frequencies, and percentages.

## Results

After the search, we retrieved 6022 records from the WHO ICTRP, 9358 preprint records and 356 records from Cochrane COVID–19 Study Register related to COVID-19. After screening those records, we included 698 records from the WHO ICTRP, 32 preprints and 65 full-texts of published articles with RCT results or protocols, which reported that they used SoC in the intervention and/or comparator group. When the SoC was used in the intervention group, the SoC was used in addition to the tested intervention, while the comparator group received only SoC.

SoC components were described in 106 (15%) of the WHO ICTRP records, 16 (50%) of preprints and 7 (11%) of included full-text manuscripts. We analyzed 737 non-overlapping sources of information. Analysis of overlap between the information sources and characteristics of each information source we analyzed are presented in detail in Supplementary file 1 (https://osf.io/he9c8/). Raw data from clinical trial registries can be found in Supplementary file 2, from preprints in Supplementary file 3, and from full-text journal articles in Supplementary file 4 (https://osf.io/he9c8/). All information sources that we identified as eligible were written in English.

### Standard of care components

Among 737 unique trials included in the analysis, 152 (21%) reported that SoC was proposed by the institutional or national authority. There were 129 (18%) trials that reported component(s) of SoC; the remaining 609 trials (82%) simply reported that they used SoC, with no further detail. Among the 129 trials that reported the components of SoC, there were 101 (78%) trials on hospitalized patients, 7 (5%) on outpatients and 7 (5%) trials where both hospitalized and outpatients were eligible. In 14 (11%) trials, the trialists did not report whether they included hospitalized patients or outpatients (Supplementary file 5).

In 129 trials that reported SoC, the number of SoC components ranged from 1 to 10. Most commonly, SoC had 2 components (in 35% of trials) or 3 components (18% of trials). There were 18 trials (14%) with five or more components of SoC (Table 1).

**Table 1:** Number of components of standard of care (SoC) used in 129 trials that described SoC components that were used

Characteristic	Results^**a**^
**Number of components**, N (%)	
1	28 (22)
2	23 (18)
3	32 (25)
4	16 (12)
5	12 (9)
6	7 (5)
7	5 (4)
8	3 (2)
9	2 (2)
10	1 (1)
**Total**	**129 (100%)**

^a^Due to rounding, numbers may not add up to 100%

Details about SoC components in included trials are presented in Supplementary file 5 (https://osf.io/he9c8/). Antiparasitics, antivirals, and antibiotics were used most commonly in SoC, in 62, 57, and 31% of the trials, respectively. Some trials used up to four different types of antivirals, antibiotics, and vitamins. Oxygen was used in 17% of the trials as a part of SoC (Table 2).

**Table 2:** Groups of interventions used in standard of care (SoC) in all trials that described the SoC (*N =* 129), and in three subgroups: trials that included outpatients (*N =* 7), both hospitalized patients and outpatients (*N =* 7) or hospitalized patients only (*N =* 101))

Intervention	N (%) and [range]^**b**^ of trials that used the intervention (***N =*** 129)	N (%) and [range]^**b**^of trials that included outpatients (***N =*** 7)	N (%) and [range]^**b**^ of trials that included both hospitalized and outpatients (***N =*** 7)	N (%) and [range]^**b**^ of trials that included hospitalized (***N =*** 101)
Antiparasitics	80 (62) [1 to 2]	4 (57) [1]	5 (71) [1 to 2]	62 (61) [1 to 2]
Antivirals	73 (57) [1 to 4]	–	2 (28) [1]	64 (63) [1 to 4]
Antibiotics	40 (31) [1 to 4]	2 (28) [1]	2 (28) [1]	34 (37) [1 to 4]
Oxygen	19 (17) [1]	–	1 (14) [1]	14 (14) [1]
Antithrombotics/anticoagulants	18 (14) [1 to 2]	–	2 (28) [1 to 2]	16 (16) [1 to 2]
Vitamins	17 (13) [1 to 4]	2 (28) [1]	3 (43) [1]	5 (5) [1 to 4]
Immunomodulatory agents	17 (13) [1 to 2]	–	1 (14) [1]	13 (13) [1 to 2]
Corticosteroids	16 (12) [1 to 2]	–	1 (14) [1]	14 (14) [1 to 2]
Analgesics/antipyretics	16 (12) [1]	4 (57) [1]	1 (14) [1]	9 (9) [1]
Other^**a**^	28 (22) [1 to 5]	1 (14) [1]	–	16 (16) [1 to 5]

^**a**^Described in detail in Supplementary file 7; examples include Antitussives, Bromhexine, Convalescent plasma, Ketamine, etc.

^**a**^Square brackets denote the range of the number of interventions used in the analyzed trials if more than one intervention from that group was used in a single trial; for example, among trials that used antiparasitics, some trials used 1 antiparasitic, others used 2 antiparasitics. Among trials that used antivirals, some trials used as many as 4 antivirals, etc.

Among trials that included outpatients, either alone or together with hospitalized patients, antiparasitics were the most commonly used type of SoC. However, in the subgroup of hospitalized patients, antivirals were the most frequently used type of SoC (Table 2).

We used 10 categories of interventions shown in Table 2 to analyze which categories and combinations of categories were used the most in trials that had described SoC components. The most commonly used SoC was a combination of antiviral(s) and antiparasitic(s). The second most common category of interventions used in SoC included only antiparasitic(s), followed by only antiviral(s) (Table 3). Categories of interventions that were used in more than one trial are shown in Table 3, while all the categories used in all 129 trials that had described SoC components are shown in a table in Supplementary file 6 (https://osf.io/he9c8/). In the 129 unique trials, the authors used 57 different categories and combinations of categories of interventions (Supplementary file 6; https://osf.io/he9c8/).

**Table 3:** Categories of interventions in the standard of care for COVID-19 (*N =* 129), shown only for categories that were used in more than one trial

Categories of interventions	N (%)
Antiviral(s) + Antiparasitic(s)	29 (22)
Antiparasitic(s)	17 (13)
Antiviral(s)	7 (5.4)
Antibiotic(s) + Antiviral(s) + Antiparasitic(s)	5 (3.9)
Antibiotic(s) + Antiparasitic(s)	5 (3.9)
Oxygen	4 (3.1)
Antiviral(s) + Antiparasitic(s) + Immunomodulating agents	3 (2.3)
Antithrombotic(s)/Anticoagulant(s)	3 (2.3)
Antiviral(s) + Immunomodulating agents	2 (1.5)
Antibiotic(s) + Antiparasitic(s) + Oxygen + Antithrombotic(s)/Anticoagulant(s) + Corticosteroid(s)	2 (1.5)
Antiviral(s) + Corticosteroid(s) + Other(s)	2 (1.5)
Other(s)	2 (1.5)
Antibiotic(s) + Antiviral(s)	2 (1.5)
Antiviral(s) + Oxygen + Immunomodulating agents	2 (1.5)
Antibiotic(s) + Antiparasitic(s) + Analgesic(s)/Antipyretic(s)	2 (1.5)

The highest number of combinations was 7 in one trial that had used the following categories of SoC: Antibiotic(s) - Antiviral(s) - Oxygen - Antithrombotic(s)/Anticoagulant(s) - Vitamin(s) - Corticosteroid(s) - Other (Supplementary file 6; https://osf.io/he9c8/). Twelve trials used combinations that included from 5 to 7 different categories (Supplementary file 6; https://osf.io/he9c8/).

Among studies including only outpatients (Table 4), or both inpatients and outpatients (Table 5), 4 combinations of interventions were used in SoC.

**Table 4:** Categories of interventions in the standard of care for COVID-19 (*N =* 7) for studies including only outpatients

Categories of interventions	N (%)
Antiparasitic(s)	1 (14)
Antibiotic(s) + Antiparasitic(s) + Analgesic(s)/antipyretic(s)	1 (14)
Antibiotic(s) + Antiparasitic(s)	1 (14)
Vitamin(s) + Analgesic(s)/antipyretic(s)	1 (14)
Vitamin(s)	1 (14)
Analgesic(s)/antipyretic(s)	1 (14)
Antiparasitic(s) + Analgesic(s)/antipyretic(s) + Other(s)	1 (14)

**Table 5:** Categories of interventions in the standard of care for COVID-19 (*N =* 7) for studies including inpatients and outpatients

Categories of interventions	N (%)
Antiparasitic(s)	2 (28)
Antiviral(s) + Antiparasitic(s) + Immunomodulating agents	1 (14)
Antithrombotic(s)/Anticoagulant(s)	1 (14)
Oxygen + Antithrombotic(s)/Anticoagulant(s) + Vitamin(s) + Corticosteroid(s) + Analgesic(s)/antipyretic(s)	1 (14)
Antibiotic(s) + Antiparasitic(s) + Vitamin(s)	1 (14)
Antibiotic(s) + Antiviral(s) + Antiparasitic(s) + Vitamin(s)	1 (14)

When looking into interventions used in SoC in each of the ten categories of interventions (Supplementary file 7; https://osf.io/he9c8/), we can see that details of these interventions were often poorly reported, including missing details about the posology, timing and method of administration in many trials. For example, one trial reported that they used “antimicrobials” within the SoC, without any further details. Another trial reported the use of “medication for pain”. Information about the SoC components was frequently partial with, for example, dose reported, but timing and the method of administration were not reported (Supplementary file 7; https://osf.io/he9c8/).

Among 372 therapies described within the SoC, three relevant pieces of information (time of administration, dose and method of administration) were not reported for 176 (47%) interventions. All three information were reported for 47 (13%) interventions. Two of the information were reported for 92 (25%) of interventions, and only single information (either time or dose or method of administration) for the remaining 57 (15%) interventions (Supplementary file 7; https://osf.io/he9c8/).

## Discussion

The study presented the first analysis of components of SoC for COVID-19 in registered and published clinical trials. The results point out that in most (82%) of the clinical trials registered and published in the first 9 months after the onset of COVID-19 that used SoC, the intervention that was labeled as SoC was not described. Among trials that did provide a description of the SoC, 60% used 3 or more components. Antiparasitics, antivirals, and antibiotics were the most commonly used interventions as components of SoC. A fifth of studies reported that SoC was determined by the regulatory government or state authorities.

We were unable to find other studies about the types of SoC used for COVID-19 in the published literature. Thus, we cannot compare our results with other similar reports.

Antiparasitics were the most commonly used single component of the SoC. Early after the emergence of COVID-19, there was much hype regarding antiparasitics, due to evidence that some of them inhibit the replication of viruses in vitro [5]. Even though some of the early studies regarding the efficacy of antiparasitics for COVID-19 appeared to be promising, very soon reports about problems with those studies emerged, and some of them were retracted [6].

Many studies and a Cochrane review provided evidence that there was no benefit in all stages of COVID-19 nor mortality benefits from the use of chloroquine and hydroxychloroquine [7, 8]. Even more, the use of chloroquine and hydroxychloroquine was associated with higher mortality and other adverse events [9]. Another Cochrane review concluded that the reliable evidence does not support the use of ivermectin for treating or preventing COVID-19 outside of well-designed RCTs [10].

Antivirals were the second most used single component of SoC for COVID-19. Early studies have shown that the repurposed medication of combination lopinavir/ritonavir, used to treat HIV infection, might play a role in improving outcomes by severe patients [11]. Subsequent research has shown that there is no benefit in mortality, duration of hospital stay, or risk of progressing to invasive mechanical ventilation or death by using lopinavir/ritonavir [12, 13].

Antibiotics were the third most commonly used category of interventions in the SoC. This can appear counterintuitive as COVID-19 is a viral disease, and it could be anticipated that only a few COVID-19 patients would have bacterial co-infection. Adebisi et al. reviewed national treatment guidelines of 10 African countries to explore the use of antibiotics in COVID-19 management; they found that 17 different antibiotics were recommended for use in treating COVID-19, some countries even for the management of mild COVID-19 [14]. Literature analysis also showed the heavy use of antibiotics in the clinical management of COVID-19, warning about the consequences of this repurposing, impending worsening of antibiotic resistance crisis and calling for the strengthening of antibiotic stewardship [15].

It is worth emphasizing that in this study, due to relatively few studies available for the main analysis of the SoC components, we did not conduct a subgroup analysis based on the stage of the disease. COVID-19 can be classified into several stages: mild, moderate, and critical stage [16]. It is presumed that the disease stage would influence the components of SoC. For example, one of the most common components of SoC was oxygen. While it could be anticipated that oxygen would be used for more advanced stages of COVID-19, it has been reported that there are different approaches to oxygen therapy in different settings. Mansab et al. analyzed the association of oxygen and mortality in COVID-19 pneumonia in a comparative analysis of supplemental oxygen policies and health outcomes across 26 countries. They found that national guidelines for starting supplemental oxygen in COVID-19 patients differed significantly between the analyzed countries. Combined, the target SpO2 for the commencement of oxygen and target oxygen saturation for ongoing treatment varied from 90 to 98%. In nations that used a conservative oxygen strategy, they found an association with higher national mortality rates [17].

Some components of SoC were likely motivated by reports about clinical abnormalities observed in the COVID-19 patients. For example, early studies have shown that coagulopathy is a common abnormality in COVID-19 disease [18]. Di Minno et al. have shown that the prevalence of venous thromboembolism (VTE) is 30%, deep vein thrombosis (DVT) was reported for 20%, and pulmonary embolism (PE) was reported for 18% in COVID-19 patients [19]. Despite such a high risk of thromboembolism, with a potentially fatal outcome, anticoagulants were used only in 18 (14%) trials that had described SoC. In 3 trials, anticoagulants were the only intervention category used in SoC, but in other trials, it was used in combination with other interventions.

Srivastava et al. conducted a meta-analysis about the use of acetylsalicylic acid (ASA) in preventing thromboembolism and concluded that the use of ASA is useful in reducing the mortality of COVID-19 patients [20]. Chow et al. showed in an observational study that ASA use among hospitalized COVID- 19 patients is associated with decreased mechanical ventilation, intensive care unit admission, and in-hospital mortality [21]. In our sample, we did not find any study that used ASA as SoC for anticoagulation and prevention of thromboembolism, nor in the analgesic/antipyretic category. The usefulness of ASA for anticoagulation, thromboembolism prevention, analgesic, and antipyretic use in COVID-19 patients remains to be further evaluated by future studies.

Current medical literature regarding vitamin support in the treatment and prevention of COVID-19 is dominated by studies about vitamin D [22–24]. Vitamin support was used as a part of SoC in 11 trials in our study; most of them used vitamin C (8 studies), while vitamin D was a part of SoC in only 4 trials. Vitamin D is used with the expectation that it would support immune response during respiratory viral infections [25]. However, a Cochrane review found that there is currently insufficient evidence to determine the benefits and harms of vitamin D supplementation as a treatment of COVID-19. Furthermore, evidence for its effectiveness was very uncertain, and limited safety information was available [26].

Corticosteroids were used in only 12% of the trials that described SoC. It is possible that the decision to use corticosteroids as a part of SoC was determined by the severity of COVID-19. However, corticosteroids have also been tested in non-oxygen requiring COVID-19 patients since the emergence of SARS-SoV-2, with the results now showing that they can be more detrimental than beneficial [27]. A Cochrane review found some benefits of corticosteroids in hospitalized patients [28].

Finally, even though we found 18% of trials that reported components of SoC, it needs to be emphasized that reporting in those trials was frequently very poor, with details about the posology, timing and method of administration often missing. Research that is poorly reported is considered research waste. To be replicable, clinical trials need to be transparently reported, and providing details about interventions is essential. This is also important for many other reasons, such as more accurate risk-benefit assessment, adherence to reporting guidelines, ethics and future research. Authors of future trials need to transparently report their interventions in all reports about the trial, including the study registrations, study protocols and full research reports.

Determining the SoC in an emergent disease is of utmost importance; however, the genuine SoC needs to be evidence-based. Challenges associated with research during such an emergent disease are acknowledged [29]. However, our study indicates that many experimental, i.e. investigational interventions, were called SoC in early COVID-19 trials, even though their risk/benefit profile in targeted patients was unknown. Therefore, we recommend that the term SoC should not be used lightly in reports about interventions for emergent diseases.

It is important to emphasize that this was not a study that aimed to determine the efficacy and safety of any type of SoC for COVID-19. We are aware that many interventions used early in the COVID-19 pandemic to treat patients can be considered experimental (i.e. investigational) as the disease was previously unknown. Instead, our intention was to analyze which interventions the trialists declared as SoC. The trialists did not have to use the term SoC when describing the therapies they decided to give to their patients in the trial. The term SoC implies that something is the standard, i.e. usual therapy, in a certain setting. We consider that in the early stage of the pandemic, there could be no SoC in the real sense, since the disease was new. Thus, it was curious to us that so many trialists opted to use the term SoC. The wide heterogeneity of the SoC found in the included studies showcases all kinds of experimental/investigational approaches that were tested in the trial setting when treating COVID-19 patients.

As a potential limitation of the study, we could have missed some overlap between the analyzed information sources, despite our best efforts to avoid that. Also, we did not attempt to analyze protocols uploaded as a supplementary appendix (in published articles or preprints), or shared publicly in trial registries such as ClinicalTrials.gov.

## Conclusion

Most RCTs (82%) about treatment for COVID-19 that were registered or published in the first 9 months of the pandemic did not describe the “standard of care” they used. Many of the SoC interventions for COVID-19 have, by now, been shown as ineffective or even detrimental.

## Supplementary Information

**Additional file 1 Supplementary file 1.** Analysis of overlap between the information sources and characteristics of reports found in each information source (Available at Open Science Framework: https://osf.io/he9c8/)

**Additional file 2 Supplementary file 2.** Raw data from clinical trial registries (Available at Open Science Framework: https://osf.io/he9c8/)

**Additional file 3 Supplementary file 3.** Raw data from preprints (Available at Open Science Framework: https://osf.io/he9c8/)

**Additional file 4 Supplementary file 4.** Raw data from full-text journal manuscripts (Available at Open Science Framework: https://osf.io/he9c8/)

**Additional file 5 Supplementary file 5.** Components of the standard of care in unique clinical trials (without overlapping information sources) (Available at Open Science Framework: https://osf.io/he9c8/)

**Additional file 6 Supplementary file 6.** Categorization of the standard of care used in analyzed trials (Available at Open Science Framework: https://osf.io/he9c8/)

**Additional file 7 Supplementary file 7.** Interventions used in each category of the standard of care (Available at Open Science Framework: https://osf.io/he9c8/)

## Data Availability

All raw data collected within this study are available in the supplementary files of this manuscript.
