# Supplementary material for: Standard of care for COVID-19 in randomized clinical trials registered in trial registries and published in preprint servers and scholarly journals: a cross-sectional study
Source: BMC Med Res Methodol. 2022 Jun 17;22:173. doi: 10.1186/s12874-022-01646-1 (PMC9205140; doi:10.1186/s12874-022-01646-1)
Supplement: Supplementary file 1 — Additional file 1 Supplementary file 1. Analysis of overlap between the information sources and characteristics of reports found in each information source (Available at Open Science Framework: https://osf.io/he9c8/) [file 12874_2022_1646_MOESM1_ESM.doc]

**Supplementary file 1. Analysis of overlap between the information sources and characteristics of reports found in each information source**

***Analysis of overlap between different information sources***

**Methods**: For full texts that were registered in multiple clinical trial registries, we counted these multiple registrations as one unit of analysis. For trials with multiple sources of information (one or more registrations, preprint, full-text), we included information from the most informative source, which was defined as the one with the most elaborate description of SoC. If we found discrepancies in the description of SoC between the information sources of a single trial, we reported them. After analysis of overlapping different information sources, we analyzed overlap between trials found only in registries, to account for trials registered in multiple registries. For this analysis, we compared titles of registered trials and identified identical titles.

**Results**: Analysis of overlapping studies among the three analyzed information sources found that among studies described in 65 journal articles, 26 were found in the included trial registrations, 5 were published as an included preprint, and 2 were found both in the trial registry and a preprint server.

Among trial registrations, we found 12 unique studies with multiple entries (29 entries for 12 studies), which were registered multiple times (2-5) in different trial registries, and were not found in other categories of overlap.

Among 32 preprints, 9 were found in the included trial registrations, but none of the preprints matched our included full texts. Description of overlap between the analyzed information sources is shown in Table S1.

**Table S1**. Sources of analyzed studies

|  | **Information source** | **N** |
| --- | --- | --- |
|  | RCTs that were found after deduplication in registries | 681 |
|  | RCTs that were not found as preprints nor in full text | 645 |
|  | Preprints that were not found in registries or full-text journal publications | 17 |
|  | Full-text journal publications that were not found in registries or preprint | 36 |
|  | Overlap: registries+preprints+full-text | 2 |
|  | Overlap: registries+preprints, without full-text | 9 |
|  | Overlap: registries+full-text that is not in the preprint | 24 |
|  | Overlap: Preprints+full-text that is not in the registry | 3 |
|  | **Total unique studies (sum of 1, 3, 4 i 8)** | **737** |

*Characteristics of information sources*

Most of the registered trial protocols were registered on Clinicaltrials.gov (29%), half were in the status “Recruiting”, target sample sizes ranged from 10 to 100,000 participants. The most common sponsor countries were Iran (25%), China (15%) and India (14%). In the majority of those records (75%) SoC was used in both intervention and comparator arms (Table S2).

**Table S**2. Characteristics of included registered trial protocols (N=698)

| **Characteristic** | **Results*** |
| --- | --- |
| **Registry**, N (%)  Clinicaltrials.gov  En.irct.ir  [www.chictr.org.cn](http://www.chictr.org.cn/)  www.ctri.nic.in  clinicaltrialsregister.eu | 198 (29)  169 (24)  95 (14)  94 (13)  88 (13) |
| **Recruitment status**, N (%)  Authorised  Not recruiting  Recruiting | 82 (12)  273 (40)  340 (49) |
| **Sponsor country**, N (%)  China  India  Iran  Spain  United States | 106 (15)  99 (14)  174 (25)  37 (5)  53 (8) |
| **Target size, range** | 10-100 000 |
| **Arm in which the standard of care was used****, N (%)  Both  Comparator  Intervention  Unclear | 521 (75)  169 (25)  2 (0.3)  6 (0.8) |

*Five most common categories were presented

**Regardless if the detailed description of the standard of care was reported

Among preprints, for the majority (72%), it was not mentioned whether the study protocol was published. In all of the preprints, the authors reported registration in a clinical trial registry. The most common countries of a sponsor for studies described in preprints were China (22%), Brasil (13%) and UK (9.4%), while for 22%, information about the sponsor was not reported. Participant target size ranged from 17 to 9355. For most preprints (88%), SoC was used both in the intervention and comparator arms (Table S3).

**Table S**3. Characteristics of included preprints

| **Characteristic** | **Results*** |
| --- | --- |
| **Study protocol published**, N (%)  Not declared in full text  Published in the appendix  Published online | 23 (72)  2 (6)  7 (22) |
| **Reported registration in a clinical trial registry**, N (%)  Yes  No | 32 (100)  0 (0) |
| **Sponsor country**, N (%)  Brasil  China  India  UK  USA  Not declared | 4 (12)  7 (22)  2 (6)  3 (9)  2 (6)  7 (22) |
| **Target size**, range | 17 - 9355 |
| **Arm in which the standard of care was used****, N (%)  Both  Comparator  Unclear | 28 (88)  3 (9)  1 (3) |

*Five most common categories were presented

**Regardless if the detailed description of the standard of care was reported

Among full-text articles, most of the publications were published in the journal Trials (52%). Among 26 journals, 18 (69%) had a JIF, which ranged from 0.873 to 74.70. There were 63% articles that reported study protocols, and the remaining 37% reported study results. In the 60 articles, the country of the sponsor was reported; most commonly China (28%), Iran (9.2%), Spain (7.7%), UK (7.7%) and Brasil (7.7%). The total number of participants (planned or included) ranged from 18 to 11303. Standard of care was used both in the intervention and comparator group in 94% of the articles (Table S4).

**Table S**4. Characteristics of included full-text journal articles

| **Characteristic** | **Results*** |
| --- | --- |
| **Journal name,** N (%)  J Antimicrob Chemother  JAMA  N Engl J Med  Encephale  Trials | 2 (3)  3 (5)  3 (5)  2 (3)  34 (52) |
| **Journal Impact Factor**, range | 0.873 to 74.70 |
| **Content of the article**, N (%)  Protocol  Results | 41 (63)  24 (37) |
| **Sponsor country**, N (%)  China  Iran  UK  Brasil  Spain | 18 (28)  6 (9)  5 (8)  5 (8)  5 (8) |
| **Number of participants planned**, range | 18 - 11303 |
| **Arm in which the standard of care was used****, N (%)  Both  Comparator  Intervention | 61 (94)  4 (6)  0 (0) |

*Five most common categories were presented

**Regardless if the detailed description of the standard of care was reported
