# Supplementary material for: Standard of care for COVID-19 in randomized clinical trials registered in trial registries and published in preprint servers and scholarly journals: a cross-sectional study
Source: BMC Med Res Methodol. 2022 Jun 17;22:173. doi: 10.1186/s12874-022-01646-1 (PMC9205140; doi:10.1186/s12874-022-01646-1)
Supplement: Supplementary file 6 — Additional file 6 Supplementary file 6. Categorization of the standard of care used in analyzed trials (Available at Open Science Framework: https://osf.io/he9c8/) [file 12874_2022_1646_MOESM6_ESM.docx]

**Supplementary table 6. Categories of interventions in the standard of care for COVID-19 (N=129)**

In 8 studies, only one category was used; the Other(s)s used from 2 to 7 combinations of different categories of interventions

| **Categories of interventions** | **N (%)*** |
| --- | --- |
| Antiviral(s) - Antiparasitic(s) | 29 (22) |
| Antiparasitic(s) | 17 (13) |
| Antiviral(s) | 7 (5.4) |
| Antibiotic(s) - Antiviral(s) - Antiparasitic(s) | 5 (3.9) |
| Antibiotic(s) - Antiparasitic(s) | 5 (3.9) |
| Oxygen | 4 (3.1) |
| Antiviral(s) - Antiparasitic(s) - Immunomodulating agents | 3 (2.3) |
| Antithrombotic(s)/Anticoagulant(s) | 3 (2.3) |
| Antiviral(s) - Immunomodulating agents | 2 (1.5) |
| Antibiotic(s) - Antiparasitic(s) - Oxygen - Antithrombotic(s)/Anticoagulant(s) - Corticosteroid(s) | 2 (1.5) |
| Antiviral(s) - Corticosteroid(s) - Other(s) | 2 (1.5) |
| Other(s) | 2 (1.5) |
| Antibiotic(s) - Antiviral(s) | 2 (1.5) |
| Antiviral(s) - Oxygen - Immunomodulating agents | 2 (1.5) |
| Antibiotic(s) - Antiparasitic(s) - Analgetic(s)/antipiretic(s) | 2 (1.5) |
| Antibiotic(s) - Antiparasitic(s) - Vitamin(s) | 1 (0.7) |
| Vitamin(s) - Analgetic(s)/antipiretic(s) | 1 (0.7) |
| Antibiotic(s) - Antiviral(s) - Antiparasitic(s) - Oxygen - Antithrombotic(s)/Anticoagulant(s) - Corticosteroid(s) | 1 (0.7) |
| Antibiotic(s) - Antithrombotic(s)/Anticoagulant(s) - Corticosteroid(s) - Analgetic(s)/antipiretic(s) - Other(s) | 1 (0.7) |
| Antibiotic(s) - Antiviral(s) - Analgetic(s)/antipiretic(s) - Other(s) | 1 (0.7) |
| Antibiotic(s) - Antiviral(s) - Antiparasitic(s) - Oxygen - Analgetic(s)/antipiretic(s) - Other(s) | 1 (0.7) |
| Antibiotic(s) - Antiviral(s) - Vitamin(s) - Other(s) | 1 (0.7) |
| Antibiotic(s) - Antiviral(s) - Antiparasitic(s) - Oxygen - Antithrombotic(s)/Anticoagulant(s) - Analgetic(s)/antipiretic(s) | 1 (0.7) |
| Antiviral(s) - Antiparasitic(s) - Antithrombotic(s)/Anticoagulant(s) | 1 (0.7) |
| Antiparasitic(s) - Antithrombotic(s)/Anticoagulant(s) | 1 (0.7) |
| Antibiotic(s) - Antiparasitic(s) - Corticosteroid(s) - Analgetic(s)/antipiretic(s) - Other(s) | 1 (0.7) |
| Immunomodulating agents | 1 (0.7) |
| Oxygen - Antithrombotic(s)/Anticoagulant(s) - Vitamin(s) - Corticosteroid(s) - Analgetic(s)/antipiretic(s) | 1 (0.7) |
| Antibiotic(s) - Antiviral(s) - Oxygen - Antithrombotic(s)/Anticoagulant(s) - Vitamin(s) - Corticosteroid(s) - Other(s) | 1 (0.7) |
| Antibiotic(s) - Antiviral(s) - Antiparasitic(s) - Vitamin(s) - Analgetic(s)/antipiretic(s) | 1 (0.7) |
| Antibiotic(s) - Antithrombotic(s)/Anticoagulant(s) - Corticosteroid(s) - Immunomodulating agents | 1 (0.7) |
| Antibiotic(s) - Antiviral(s) - Antiparasitic(s) - Other(s) | 1 (0.7) |
| Antibiotic(s) - AB - Antiparasitic(s) | 1 (0.7) |
| Antiviral(s) - Other(s) | 1 (0.7) |
| Antibiotic(s) - Antiviral(s) - Immunomodulating agents | 1 (0.7) |
| Antiviral(s) - Antithrombotic(s)/Anticoagulant(s) | 1 (0.7) |
| Vitamin(s) - Analgetic(s)/antipiretic(s) - Other(s) | 1 (0.7) |
| Antibiotic(s) - Oxygen - Antithrombotic(s)/Anticoagulant(s) - Vitamin(s) | 1 (0.7) |
| Antibiotic(s) - Antiviral(s) - Antiparasitic(s) - Immunomodulating agents | 1 (0.7) |
| Antibiotic(s) - Antiparasitic(s) - Oxygen | 1 (0.7) |
| Vitamin(s) | 1 (0.7) |
| Antibiotic(s) - Antiviral(s) - Antiparasitic(s) - Vitamin(s) | 1 (0.7) |
| Oxygen - Analgetic(s)/antipiretic(s) | 1 (0.7) |
| Analgetic(s)/antipiretic(s) | 1 (0.7) |
| Antibiotic(s) - Antiparasitic(s) - Immunomodulating agents | 1 (0.7) |
| Antiviral(s) - Corticosteroid(s) | 1 (0.7) |
| Antibiotic(s) - Vitamin(s) - Other(s) | 1 (0.7) |
| Oxygen - Antithrombotic(s)/Anticoagulant(s) | 1 (0.7) |
| Antibiotic(s) - Antiparasitic(s) - Antithrombotic(s)/Anticoagulant(s) - Corticosteroid(s) | 1 (0.7) |
| Antiviral(s) - Corticosteroid(s) - Immunomodulating agents | 1 (0.7) |
| Antiviral(s) - Immunomodulating agents - Other(s) | 1 (0.7) |
| Antithrombotic(s)/Anticoagulant(s) - Corticosteroid(s) - Immunomodulating agents | 1 (0.7) |
| Antibiotic(s) - Antiviral(s) - Oxygen - Corticosteroid(s) - Other(s) | 1 (0.7) |
| Antiparasitic(s) - Analgetic(s)/antipiretic(s) - Other(s) | 1 (0.7) |
| Antibiotic(s) - Antiparasitic(s) - Vitamin(s) - Analgetic(s)/antipiretic(s) - Other(s) | 1 (0.7) |
| Antibiotic(s) - Antiviral(s) - Antiparasitic(s) - Oxygen | 1 (0.7) |
| Analgetic(s)/antipiretic(s) - Other(s) | 1 (0.7) |
| **Total** | **129 (100)** |

*The numbers may not add to 100% due to rounding
