# Supplementary material for: Standard of care for COVID-19 in randomized clinical trials registered in trial registries and published in preprint servers and scholarly journals: a cross-sectional study
Source: BMC Med Res Methodol. 2022 Jun 17;22:173. doi: 10.1186/s12874-022-01646-1 (PMC9205140; doi:10.1186/s12874-022-01646-1)
Supplement: Supplementary file 7 — Additional file 7 Supplementary file 7. Interventions used in each category of the standard of care (Available at Open Science Framework: https://osf.io/he9c8/) [file 12874_2022_1646_MOESM7_ESM.docx]

**Supplementary table 7. List of all described components of SoC as they appeared and declared in list of studies**

**Table 1. Antiparasitics**

| **therapy name** | **period** | **dose** | **regimen** |
| --- | --- | --- | --- |
| Amoxacillin/clavulanate | not declared | 100 mg/kg/day | not specified |
| Antibacterial agents | not declared | not declared | not declared |
| Antibiotics | not declared | not declared | not declared |
| Antibiotics | not declared | not declared | not declared |
| Antibiotics | not declared | not declared | not declared |
| Antibiotics (Azithromycin or Doxycycline) | not declared | not declared | not declared |
| Antibiotics (Azithromycin/Ceftriaxone) | not declared | not declared | not declared |
| Azithromycin | 5 days | 500 mg | not declared |
| Azithromycin | 5 days | 500 mg | not declared |
| Azithromycin | not declared | 500 mg | oral |
| Azithromycin | 5 days | 500 mg | oral |
| Azithromycin | not declared | 500 mg | not declared |
| Azithromycin | 3 days | 500 mg | not declared |
| Azithromycin | not declared | not declared | not declared |
| Azithromycin | not declared | 250 mg | not declared |
| Azithromycin | 5 days | Day 1:500 mg/daily day 2-5: 250mg/daily | not declared |
| Azithromycin | not declared | 1 g first day then 500 mg/day | not declared |
| Azithromycin | 5 days | 500 mg/daily | not declared |
| Azithromycin | not declared | 500 mg/daily | not declared |
| Azithromycin | not declared | not declared | not declared |
| Azithromycin | 5 days | Day 1:500 mg/daily day 2-5: 250mg/daily | not declared |
| Azithromycin | 7 days | Day 1:500 mg/daily day 2-6: 250mg/daily | not declared |
| Azithromycin | 5 days | 500 mg/daily | oral |
| Azithromycin | 5 days | 500 mg/daily | oral |
| Azithromycin | not declared | not declared | not declared |
| Azithromycin | up to 7 days | 500 mg/daily | oral |
| Azithromycin | not declared | not declared | not declared |
| Azithromycin | not declared | 500 mg/daily | not declared |
| Azithromycin | not declared | not declared | not declared |
| Azithromycin | not declared | not declared | not declared |
| Azithromycin | not declared | not declared | not declared |
| Azithromycin | 5 days | 500 mg/daily-1.day then 25o mg/daily | not declared |
| Azithromycin | not declared | not declared | not declared |
| Azithromycin | not declared | 500 mg/daily | oral |
| Azithromycin + Amoxicillin/Clavulanic acid | not declared | not declared | not declared |
| Azithromycine | not declared | not declared | not declared |
| Azythromycin | not declared | not declared | not declared |
| Ceftriaxone | not declared | not declared | not declared |
| Ceftriaxone | 7 days | 2 g/daily | not declared |
| Ceftriaxone | not declared | not declared | not declared |
| Ceftriaxone | 5 days | 2 g/daily | i.v. |
| Clarithromycin | 7-14 days | 1000 mg/daily | not specified |
| Doxycycline | 5 days | 200 mg | oral |
| Doxycycline | 10 days | 200 mg daily | not declared |
| Empiric Antimicrobials | not declared | not declared | not declared |
| Empirical Antibiotic Therapy | not declared | not declared | not declared |
| Levofloxacin | 5 days | 750 mg/daily or 500mg/daily | not declared |
| Levofloxacin | not declared | 750 mg/daily | not declared |
| Macrolide | not declared | not declared | not declared |
| Macrolides | not declared | not declared | not declared |
| Piperacillin + Tazobactam | 5 days | 13.5 mg | i.v. |

**Table 2. Antivirals**

| **therapy name** | **period** | **dose** | **regimen** |
| --- | --- | --- | --- |
| Antiretroviral therapy Lopinavir/Ritonavir or Darunavir/Cobicistat is permitted | not declared | not declared | not declared |
| Antiviral agents | not declared | not declared | not declared |
| Antiviral treatment | not declared | not declared | not declared |
| Antivirals | not declared | not declared | not declared |
| Antivirals | not declared | not declared | not declared |
| Antivirals | not declared | not declared | not declared |
| Arbidol (Umifenovir) | not declared | not declared | not declared |
| Arbidol (Umifenovir) | not declared | 600 mg/daily | oral |
| Atazanavir | 7 days and up to 14 days. | 400 mg/daily | not declared |
| Atazanavir | 7 days and up to 14 days. | 400 mg/daily | not declared |
| Atazanavir/Ritonavir | 7-10 days | 300 mg/100 mg daily | not declared |
| Atazanavir/Ritonavir | 14 days | 300/100 mg/daily | not declared |
| Atazanavir/Ritonavir | not declared | not declared | not declared |
| Atazanavir/Ritonavir | not declared | not declared | not declared |
| Atazanavir/Ritonavir | 7 days and up to 14 days. | 300 mg/100mg | oral |
| Atazanavir/Ritonavir | 7 days and up to 14 days. | 300 mg/100mg | oral |
| Darunavir | not declared | 800 mg/daily | not declared |
| Darunavir/Cobicistat | not declared | 800/150 mg/daily | not declared |
| Darunavir/Cobicistat | not declared | 800/150 mg, once daily | oral |
| Darunavir/Ritonavir | not declared | not declared | not declared |
| Kaletra (Lopinavir/Ritonavir) | not declared | 200 mg/800 mg daily | not declared |
| Kaletra (Lopinavir/Ritonavir) | 10 days | 800 mg/daily | not declared |
| Kaletra (Lopinavir/Ritonavir) | not declared | not declared | not declared |
| Kaletra (Lopinavir/Ritonavir) | not declared | not declared | not declared |
| Kaletra (Lopinavir/Ritonavir) | not declared | not declared | not declared |
| Kaletra (Lopinavir/Ritonavir) | not declared | not declared | not declared |
| Kaletra (Lopinavir/Ritonavir) | not declared | not declared | not declared |
| Kaletra (Lopinavir/Ritonavir) | not declared | not declared | not declared |
| Kaletra (Lopinavir/Ritonavir) | 5 days | 200 mg/800 mg daily | oral |
| Kaletra (Lopinavir/Ritonavir) | 5 days | 400 mg/400 mg | oral |
| Kaletra (Lopinavir/Ritonavir) | 7 days | 800 mg/daily | oral |
| Kaletra (Lopinavir/Ritonavir) | 14 days | not declared | oral |
| Kaletra tablet (Lopinavir/Ritonavir) | 5 - 14 days | 200/50 mg, 2 tablets every 12 hours | oral |
| Kaletra tablets (Lopinavir/Ritonavir) | 7 days and up to 14 days. | 200/800 mg/daily | oral |
| Kaletra tablets (Lopinavir/Ritonavir) | 7 days and up to 14 days. | 200/800 mg/daily | oral |
| Lopinavir | not declared | 400 mg | oral |
| Lopinavir/Ritonavir | 7 days | 800 mg/200 mg | i.v. |
| Lopinavir/Ritonavir | 10 days | 100 mg-400 mg | not declared |
| Lopinavir/Ritonavir | 7 days | 200 mg/50 mg | not declared |
| Lopinavir/Ritonavir | not declared | 400 mg /100 mg daily | not declared |
| Lopinavir/Ritonavir | not declared | 400 mg/100 mg daily | not declared |
| Lopinavir/Ritonavir | 7-10 days | 800/200 mg | not declared |
| Lopinavir/Ritonavir | 5 days | every 12 hours, 2 tablets 50 mg/200 mg | not declared |
| Lopinavir/Ritonavir | until the patient's clinical symptoms improve | lopinavir (200 mg/daily) – ritonavir (800 mg) | not declared |
| Lopinavir/Ritonavir | 14 days | not declared | not declared |
| Lopinavir/Ritonavir | not declared | not declared | not declared |
| Lopinavir/Ritonavir | not declared | not declared | not declared |
| Lopinavir/Ritonavir | not declared | not declared | not declared |
| Lopinavir/Ritonavir | not declared | not declared | not declared |
| Lopinavir/Ritonavir | not declared | not declared | not declared |
| Lopinavir/Ritonavir | not declared | not declared | not declared |
| Lopinavir/Ritonavir | not declared | not declared | not declared |
| Lopinavir/Ritonavir | not declared | not declared | not declared |
| Lopinavir/Ritonavir | not declared | not declared | not declared |
| Lopinavir/Ritonavir | not declared | not declared | not declared |
| Lopinavir/Ritonavir | not declared | not declared | not declared |
| Lopinavir/Ritonavir | not declared | not declared | not declared |
| Lopinavir/Ritonavir | not declared | not declared | not declared |
| Lopinavir/Ritonavir | not declared | not declared | not declared |
| Lopinavir/Ritonavir | not declared | 1600 mg/400 mg | oral |
| Lopinavir/Ritonavir | 14 days | 200 mg/50 mg twice daily | oral |
| Lopinavir/Ritonavir | 10-14 days | 800 mg/200 mg | oral |
| Lopinavir/Ritonavir | 14 days | 800 mg/200 mg | oral |
| Lopinavir/Ritonavir | not declared | 800 mg/200 mg | oral |
| Lopinavir/Ritonavir | 10 days | 800 mg/200 mg daily | oral |
| Lopinavir/Ritonavir | 14 days | 800 mg/200 mg daily | oral |
| Lopinavir/Ritonavir | 7-14 days | 800/200 mg | oral |
| Lopinavir/Ritonavir | not declared | 800/200 mg | oral |
| Lopinavir/Ritonavir | not declared | 800/200 mg/daily | oral |
| Lopinavir/Ritonavir | not declared | not declared | oral |
| Oseltamivir | 5-10 days | 150 mg/daily | not declared |
| Oseltamivir | 5 days | 150 mg/daily | not declared |
| Oseltamivir | 5 days | 300 mg/daily | not declared |
| Oseltamivir | 10 days | 300 mg/daily | not declared |
| Oseltamivir | not declared | not declared | not declared |
| Oseltamivir | 14 days | 150 mg/daily | oral |
| Oseltamivir | not declared | 150 mg/daily | oral |
| Oseltamivir | not declared | not declared | oral |
| Oseltamivir | not declared | not declared | not declared |
| Remdesivir | not declared | 200 mg in.v on day 1, followed by a 100 mg | i.v. |
| Remdesivir | not declared | not declared | not declared |
| Remdesivir | not declared | not declared | not declared |
| Remdesivir | not declared | not declared | not declared |
| Remdesivir | not declared | not declared | not declared |
| Remdesivir | not declared | not declared | not declared |
| Remdesivir | not declared | not declared | not declared |
| Remdesivir | not declared | not declared | not declared |
| Remdesivir | not declared | not declared | not declared |
| Remdesivir | not declared | not declared | not declared |
| Remdisivir | not declared | not declared | not declared |
| Ribavirin | not declared | not declared | not declared |
| Ribavirin | not declared | 2400 mg/daily | not declared |
| Ribavirin | not declared | not declared | not declared |
| Ribavirin | not declared | not declared | not declared |
| Ribavirin | not declared | the first dose for adults 4g, 8 hours the next day, 1.2g each time, or 8mg/kg iv. once every 8 hours | not declared |
| Ritonavir | not declared | 100 mg | oral |
| Ritonavir | not declared | 100 mg/daily | oral |
| Siltamivir | not declared | 150 mg/daily | not declared |
| Tamiflu (Ostelamivir) | not declared | not declared | not declared |
| Umifenovir | not declared | not declared | not declared |

**Table 3. Antibiotics**

| **therapy name** | **period** | **dose** | **regimen** |
| --- | --- | --- | --- |
| Amoxacillin/clavulanate | not declared | 100 mg/kg/day | not specified |
| Antibacterial agents | not declared | not declared | not declared |
| Antibiotics | not declared | not declared | not declared |
| Antibiotics | not declared | not declared | not declared |
| Antibiotics | not declared | not declared | not declared |
| Antibiotics (Azithromycin or Doxycycline) | not declared | not declared | not declared |
| Antibiotics (Azithromycin/Ceftriaxone) | not declared | not declared | not declared |
| Azithromycin | 5 days | 500 mg | not declared |
| Azithromycin | 5 days | 500 mg | not declared |
| Azithromycin | not declared | 500 mg | oral |
| Azithromycin | 5 days | 500 mg | oral |
| Azithromycin | not declared | 500 mg | not declared |
| Azithromycin | 3 days | 500 mg | not declared |
| Azithromycin | not declared | not declared | not declared |
| Azithromycin | not declared | 250 mg | not declared |
| Azithromycin | 5 days | Day 1:500 mg/daily day 2-5: 250mg/daily | not declared |
| Azithromycin | not declared | 1 g first day then 500 mg/day | not declared |
| Azithromycin | 5 days | 500 mg/daily | not declared |
| Azithromycin | not declared | 500 mg/daily | not declared |
| Azithromycin | not declared | not declared | not declared |
| Azithromycin | 5 days | Day 1:500 mg/daily day 2-5: 250mg/daily | not declared |
| Azithromycin | 7 days | Day 1:500 mg/daily day 2-6: 250mg/daily | not declared |
| Azithromycin | 5 days | 500 mg/daily | oral |
| Azithromycin | 5 days | 500 mg/daily | oral |
| Azithromycin | not declared | not declared | not declared |
| Azithromycin | up to 7 days | 500 mg/daily | oral |
| Azithromycin | not declared | not declared | not declared |
| Azithromycin | not declared | 500 mg/daily | not declared |
| Azithromycin | not declared | not declared | not declared |
| Azithromycin | not declared | not declared | not declared |
| Azithromycin | not declared | not declared | not declared |
| Azithromycin | 5 days | 500 mg/daily-1.day then 25o mg/daily | not declared |
| Azithromycin | not declared | not declared | not declared |
| Azithromycin | not declared | 500 mg/daily | oral |
| Azithromycin + Amoxicillin/Clavulanic acid | not declared | not declared | not declared |
| Azithromycine | not declared | not declared | not declared |
| Azythromycin | not declared | not declared | not declared |
| Ceftriaxone | not declared | not declared | not declared |
| Ceftriaxone | 7 days | 2 g/daily | not declared |
| Ceftriaxone | not declared | not declared | not declared |
| Ceftriaxone | 5 days | 2 g/daily | i.v. |
| Clarithromycin | 7-14 days | 1000 mg/daily | not specified |
| Doxycycline | 5 days | 200 mg | oral |
| Doxycycline | 10 days | 200 mg daily | not declared |
| Empiric Antimicrobials | not declared | not declared | not declared |
| Empirical Antibiotic Therapy | not declared | not declared | not declared |
| Levofloxacin | 5 days | 750 mg/daily or 500mg/daily | not declared |
| Levofloxacin | not declared | 750 mg/daily | not declared |
| Macrolide | not declared | not declared | not declared |
| Macrolides | not declared | not declared | not declared |
| Piperacillin + Tazobactam | 5 days | 13.5 mg | i.v. |

**Table 4. Oxygen**

| **therapy name** | **period** | **dose** | **regimen** |
| --- | --- | --- | --- |
| Oxigen therapy | 14 days | not declared | inhal. |
| Oxigen therapy | not declared | not declared | inhal. |
| Oxigen therapy | not declared | not declared | inhal. |
| Oxigen therapy | not declared | not declared | not declared |
| Oxigen therapy | not declared | not declared | not declared |
| Oxigen therapy | not declared | not declared | not declared |
| Oxigen therapy | not declared | not declared | not declared |
| Oxigen therapy | not declared | not declared | not declared |
| Oxigen therapy | not declared | not declared | not declared |
| Oxigen therapy | not declared | not declared | not declared |
| Oxigen therapy | not declared | not declared | not declared |
| Oxigen therapy | not declared | not declared | not declared |
| Oxigen therapy | not declared | not declared | not declared |
| Oxigen therapy | not declared | not declared | not declared |
| Oxigen therapy | not declared | not declared | not declared |
| Oxigen therapy | not declared | not declared | not declared |
| Oxigen therapy | not declared | not declared | not declared |
| Oxigen therapy | not declared | not declared | not declared |
| Oxigen therapy | not declared | not declared | not specified |

**Table 5. Antithrombotic/anticoagulant**

| **therapy name** | **period** | **dose** | **regimen** |
| --- | --- | --- | --- |
| Thrombosis prophylaxis | not declared | not declared | not declared |
| Anticoagulant | not declared | not declared | not declared |
| Enoxaparin (LMWH) | not declared | 1 mg/kg | not declared |
| Enoxaparin (LMWH) | not declared | not declared | not declared |
| Enoxaparin (LMWH) | not declared | 1 mg/kg per day sc | other |
| Enoxaparin (LMWH) | not declared | 40 mg | s.c |
| Heparin | until the end of hospitalization | 5000 UI | not declared |
| Heparin | not declared | not declared | not declared |
| Low-molecular weight heparin (LMWH) | not declared | not declared | not declared |
| Low-molecular weight heparin (LMWH) | not declared | not declared | not declared |
| Low-molecular weight heparin (LMWH) | not declared | not declared | not declared |
| Low-molecular weight heparin (LMWH) | not declared | not declared | not declared |
| Low-molecular weight heparin (LMWH) | not declared | not declared | not declared |
| Low-molecular weight heparin (LMWH) | not declared | not declared | not declared |
| Low-molecular weight heparin (LMWH) | not declared | not declared | not declared |
| Low-molecular weight heparin (LMWH) | not declared | 10 000 IU/daily | s.c |
| Prophylaxis for deep vein thrombosis | not declared | not declared | not declared |
| Thromboprophylaxis | not declared | not declared | not declared |
| Unfractionated heparin (UFH) | not declared | not declared | not declared |

**Table 6. Vitamin**

| **therapy name** | **period** | **dose** | **regimen** |
| --- | --- | --- | --- |
| Ascorbic acid (Vitamin C) | not declared | 2 g | not declared |
| Ascorbic acid (Vitamin C) | not declared | 3 g/daily | not declared |
| Ascorbic acid (Vitamin C) | not declared | 3g/daily | not declared |
| Ascorbic acid (Vitamin C) | not declared | not declared | not declared |
| Ascorbic acid (Vitamin C) | not declared | 1000 mg/daily | not specified |
| Ascorbic acid (Vitamin C) | not declared | 1000 mg/daily | not specified |
| Ascorbic acid (Vitamin C) | not declared | 500 mg | oral |
| Ascorbic acid (Vitamin C) | 5 days | Day 1:2000 mg daily Days 2-5: 1000 mg total daily | oral |
| Cyanocobalamin | not declared | not declared | i.v. |
| Thiamine | not declared | 400 mg | not declared |
| Thiamine | not declared | 400 mg | not declared |
| Vitamin A | not declared | not declared | not declared |
| Vitamin A | not declared | not declared | not declared |
| Vitamin D | not declared | 1 µg | not declared |
| Vitamin D | not declared | not declared | not declared |
| Vitamin D | not declared | not declared | not declared |
| Vitamin D | not declared | not declared | not declared |
| Vitamin support | 10 days | not declared | not declared |
| Vitamin support | not declared | not declared | not declared |

**Table 7. Corticosteroids**

| **therapy name** | **period** | **dose** | **regimen** |
| --- | --- | --- | --- |
| Corticosteroids | not declared | not declared | not declared |
| Dexamethasone | 10 days | 6 mg | i.v. |
| Dexamethasone | not declared | not declared | not declared |
| Dexamethasone | not declared | not declared | not declared |
| Dexamethasone | not declared | not declared | not declared |
| Dexamethasone | not declared | not declared | not declared |
| Dexamethasone | not declared | not declared | not specified |
| Dexamethasone | 7 days | 6 mg | i.v. |
| Dexamethasone | not declared | not declared | not declared |
| Dexamethasone (or other steroids) | not declared | not declared | not declared |
| Methyprednisolone | 5 days | 0.5 mg/kg | i.v. |
| Prednisolone | 5 days | 25 mg/daily | not declared |
| Prednisolone | not declared | not declared | not declared |
| Steroids | not declared | not declared | not declared |
| Steroids | not declared | not declared | not declared |
| Steroids | not declared | not declared | not declared |
| Steroids | not declared | not declared | not declared |

**Table 8. Analgetics/antipiretics**

| **therapy name** | **period** | **dose** | **regimen** |
| --- | --- | --- | --- |
| Medication for pain | not declared | not declared | not declared |
| Naproxen | 5 days | 500 mg/daily | not declared |
| Naproxen | not declared | 400 mg | not declared |
| Paracetamol (Acetaminophen) | 14 days | 2000 mg/daily | not declared |
| Paracetamol (Acetaminophen) | not declared | 4000 mg/daily | not declared |
| Paracetamol (Acetaminophen) | not declared | 650 mg/daily | not declared |
| Paracetamol (Acetaminophen) | not declared | not declared | not declared |
| Paracetamol (Acetaminophen) | not declared | not declared | not declared |
| Paracetamol (Acetaminophen) | not declared | not declared | not declared |
| Paracetamol (Acetaminophen) | not declared | not declared | not declared |
| Paracetamol (Acetaminophen) | not declared | not declared | not declared |
| Paracetamol (Acetaminophen) | not declared | 3 g | oral |
| Paracetamol (Acetaminophen) | on demand | 3 g/daily | oral |
| Paracetamol (Acetaminophen) | not declared | 500 mg | oral |
| Paracetamol (Acetaminophen) | not declared | 500 mg | oral |
| Paracetamol (Acetaminophen) | not declared | not declared | oral |

**Table 9. Immunomodulating agents**

| **therapy name** | **period** | **dose** | **regimen** |
| --- | --- | --- | --- |
| alpha 2b-IFN | not declared | not declared | not declared |
| Anakinra | not declared | not declared | not declared |
| Heberferon (recombinant human interferon alpha 2b and recombinant human interferon gamma) | 3-4 weeks | 3 million IU/3 times a week | i.m. |
| Interferon alpha | not declared | 300000-400000 IU,daily | inhal. |
| Interferon alpha | not declared | 5 milion units | inhal. |
| Interferon alpha | 14 days | not declared | inhal. |
| Interferon alpha | not declared | not declared | intranasal |
| Interferon alpha | not declared | not declared | not declared |
| interferon beta -1b | not declared | not declared | not declared |
| Interferon beta-1a | not declared | not declared | not declared |
| Interferon beta-1b | not declared | not declared | not declared |
| Recombinant human interferon alpha 2b | not declared | not declared | not declared |
| Thymosin | 14 days | 0,8 mg | s.c. |
| Tocilizumab | not declared | 8 mg/kg (maximum dose of 800 mg) iv, maximum 3 doses 8 to 12 hours apart | i.v. |
| Tocilizumab | not declared | 800 mg/daily | i.v. |
| Tocilizumab | not declared | not declared | not declared |
| Tocilizumab | not declared | not declared | not declared |

**Table 10. Other**

| **therapy name** | **period** | **dose** | **regimen** |
| --- | --- | --- | --- |
| Antitussives | not declared | not declared | not declared |
| Bromhexine | not declared | 16 mg | oral |
| Convalescent plasma | not declared | not declared | not declared |
| Convalescent plasma | not declared | not declared | not declared |
| Convalescent plasma | not declared | not declared | not declared |
| Dexmedetomidine | not declared | not declared | i.v. |
| Diphenhydramine syrup | not declared | 10 cc- cubic centimeters | oral |
| Famotidine | 5 days | 80 mg/daily | not declared |
| Immunoglobulin | not declared | not declared | not declared |
| Ketamine | not declared | not declared | i.v. |
| Midazolam | not declared | not declared | i.v. |
| Montelukast | not declared | 10 mg | oral |
| Omega-3 | not declared | 500 mg daily | not declared |
| Omega-3 | not declared | 500 mg/daily | not declared |
| Omeprazole | not declared | 20 mg | oral |
| Ondasetron | not declared | 4 mg | oral |
| Pantocid | not declared | 40 mg | oral |
| Pantoprazole | not declared | 40 mg/daily | oral |
| Propofol | not declared | not declared | i.v. |
| Saline infusion | 5 days | 10 ml/daily | i.v. |
| Selenium | not declared | not declared | not declared |
| Selenium | not declared | not declared | not declared |
| Surface temperature management | not declared | not declared | not declared |
| Zinc | not declared | 50 mg | not declared |
| Zinc | not declared | not declared | not declared |
| Zinc | not declared | not declared | oral |
